# Research on 5S rDNA, Mitochondria and Nutritional Components of *Cambaroides dauricus*

**DOI:** 10.3390/biology14091215

**Published:** 2025-09-08

**Authors:** Hanbo Liu, Xiaoyi Dong, Yude Wang, Shengwei Luo

**Affiliations:** State Key Laboratory of Developmental Biology of Freshwater Fish, College of Life Sciences, Hunan Normal University, Lushan Road, No. 36, Yuelu District, Changsha 410081, China; lhb20223015@163.com (H.L.); dxy08142018@icloud.com (X.D.)

**Keywords:** decapoda, phylogenetics, mitochondrial genome, *Cambaroides dauricus*

## Abstract

The mitochondrial genome and 5S rDNA are powerful tools for exploring and confirming species identity and understanding evolutionary trajectories. In addition, evaluating the nutritional value of *Cambaroides dauricus* by determining and analyzing the nutritional components of its muscles could provide essential data for relevant artificial breeding and food processing. In this study, for the first time, we characterized the mitochondrial genome of *Cambaroides dauricus* using Illumina sequencing technology. The results showed that the mitochondrial genome was a circular genome of 16,215 bp in length. The average sequencing depth of the mitochondrial genome was 100X. The nucleotide composition of the mitochondrial genome was 33.4% A, 39.1% T, 11.0% C and 16.5% G. Phylogenetic analyses showed that *Cambaroides dauricus* has a very close relationship with *Cambaroides wladiwostokiensis*. The results of 5S rDNA showed that the genetic relationship between *Procambarus clarkii* and *Cambaroides dauricus* is quite close. After determining the nutritional components of *Cambaroides dauricus*’ muscles with normal analysis methods, the results revealed that it has a crude protein content of 18.47 ± 0.87 per 100 milligrams, a crude fat content of 0.83 ± 0.12 per 100 milligrams, a crude ash content of 0.63 ± 0.06 per 100 milligrams and a moisture content of 79.8 ± 0.72 per 100 milligrams. There are eight essential amino acids in its protein, accounting for 41.59% of the total amino acids, and the proportion of umami amino acids is 36.27%. This study will provide a valuable basis for further studies of taxonomy, phylogenetic analyses and artificial breeding in *Cambaroides dauricus*.

## Simple Summary

The research aimed to study the characteristics of the mitochondrial genome, nutritional components, and genetic relationships of *Cambaroides dauricus*. Phylogenetic analyses showed that *Cambaroides dauricus* has a very close relationship with *Cambaroides wladiwostokiensis*. The results of 5S rDNA analysis indicated that the genetic relationship between *Procambarus clarkii* and *Cambaroides dauricus* is quite close. Additionally, the analysis of nutritional components in the muscles of *Cambaroides dauricus*’ muscles showed that it is highly nutritious value. The research results presented in this article have positive significance for the industry and genomic research on crayfish.

## 1. Introduction

Freshwater crayfish, which accord with the big food concept, are an important food source. “Freshwater crayfish” is the general term for freshwater species in the suborder crayfish of the order Decapoda, and they are widely distributed all over the world [1,2,3]. They include two families, Parastacoidea and Astacoidea, which contain four subfamilies (Parastacidae, Astacoidea, Cambaridae, Cricoidosceloside) [4,5]. The Parastacidae family consists of 16 genera, namely, Aenigmastacus, Astacoides, Astacopsis, Cherax, Engaewa, Engaeus, Euastacus, Gramastacus, Geocharax, Palaeoechinastacus, Paranephrops, Parastacoides, Parastacus, Samastacus, Virilastacus and Tennuibranchurus. The Asteriidae family consists of three genera, namely, Astacus, Austropotamobius and Pacifastacus. The Cambaricae family comprises 15 genera, namely Barbicambarus, Boucharidina, Cambarellus, Cambaroides, Cambarus, Creaserinus, Distocambarus, Fallicambaru, Faxonella, Faxonius, Hobseus, Lacunicambarus, Orconectes, Procambarus and Troglocambarus [4,6,7,8]. The Cricoidoscelosidae family has become extinct, with no existing species. The freshwater crayfish, *Cambaroides dauricus* (*C. dauricus*), belonging to Astacidea, Astacoidea, Cambaridae, is widely distributed in China, Russia and Democratic People’s Republic of Korea, and is a commercially important wild species in northeastern China [9,10,11]. *C. dauricus* mainly inhabits shallow waters such as rivers, lakes and streams with abundant aquatic plants. Studies have indicated its preference for flowing freshwater environments characterized by high dissolved oxygen and high transparency [10]. The body color of the *Cambaroides dauricus* ranges from grayish white to dark blackish brown, to olive green, and it can live for up to 8 years. *Cambaroides dauricus* not only has excellent ornamental value but is also highly nutritious [10]. *Cambaroides dauricus* possesses several advantages including high protein palatable flavor and cold resistance. It also has high value in breeding and decorating. The content of crude protein and crude fat in the muscle tissue of *Cambaroides dauricus* are approximately 18.62% and 2.13%, respectively. The liver crude fat of *Cambaroides dauricus* is from 24.71% to 29.36% [12]. A close relative of P. clarkia, it has been actively researched for its fundamental ecology and aquaculture technologies, and is more tolerant of cold temperatures, with an optimal water temperature range of 16–21 °C.

The sequence of mitochondrial genomes and the construction of genomic sequence maps represent the primary content of genomics research and serve as essential methods for elucidating genetic information [13,14]. Sequencing technologies have been used to study *Cambaroides dauricus*. Therefore, we have sequenced the whole genome of *Cambaroides dauricus* to analyze its evolutionary relationship. Based on 37 complete mitochondrial genomes of *Cambaroides dauricus*, our research can provide basic information for further phylogenetic analyses of *Cambaroides dauricus* and will enrich the genetic biodiversity.

The ribosomal RNA molecule 5S rRNA is an essential element for the synthesis of large subunits in all bacteria, archaea and eukaryotes [15,16,17,18]. The coding sequence (CDS) of 5S rDNA and the non-transcribed spacer (NTS) are crucial components of the genome [19,20]. Each of these components possesses distinct functions and characteristics [21]. Specifically, the CDS in 5S rDNA plays a pivotal role in encoding 5S rRNA [22].

There have been few studies on the *Cambaroides dauricus* breeding industry in recent years, especially concerning a nutrition ingredient analysis. Our research aimed at evaluating the nutritional value of *Cambaroides dauricus*’ muscles by measuring and analyzing their nutritional components, so that our research can provide essential data for relevant artificial breeding and food processing.

## 2. Materials and Methods

### 2.1. Mitochondrial Experiment

**Figure 1:**
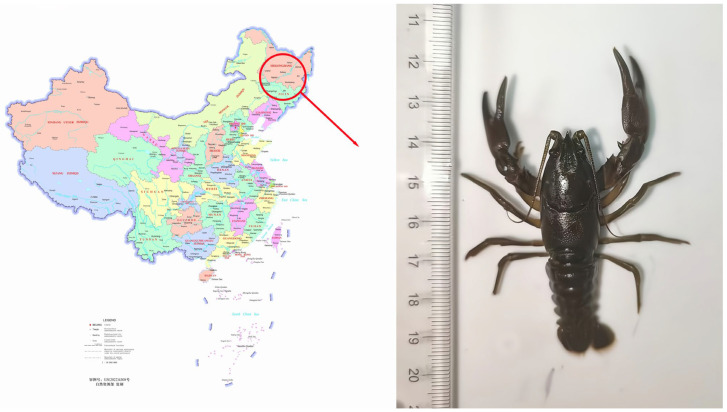
Specimens of *Cambaroides dauricus*. (The (**left**) part of the figure has been downloaded from the website http://bzdt.ch.mnr.gov.cn/ 6 February 2024; the (**right**) part is a photograph taken by Han-Bo Liu, not published in previous works.).

*Cambaroides dauricus* was collected in Changbai Mountain National Nature Reserve (127°42′55″—128°16′48″ E, 41°41′49″—42°25′18″ N, 1351 MASL), Baishan City, Jilin Province, in November 2023 (Figure 1). Before dissection, dry the surface of the *Cambaroides dauricus* with absorbent papers; then, open its shell and remove the muscle tissue and weigh it, recording the data as muscle mass. The obtained muscles were crushed through high-speed homogenization, then put into sealed bags, labeled and refrigerated at −20 °C. Determination of basic components: moisture content: GB5009.3-2003; (https://openstd.samr.gov.cn/bzgk/std/) ash content: GB5009.4-2003; crude protein content: GB5009.5-2003—Kjeldahl method; crude fat protein content: chloroform—methanol method [V(chloroform)/V(methyl alcohol) = 2:1] used for extracting prolicides, and the specific determination shall be carried out in accordance with the Folch method [23].

The *Cambaroides dauricus* samples were stored in the State Key Laboratory of Freshwater Fish Developmental Biology, College of Life Sciences, Hunan Normal University (voucher number A427-3) (https://ldbff.hunnu.edu.cn/index.htm/ 28 December 2016, Yude Wang.) Total *Cambaroides dauricus* DNA was extracted from the muscle tissue using a tissue genomic DNA extraction kit (Tiangen, Beijing, China). After DNA detection was qualified, a DNA sequencing library with an insert fragment of 350 bp was constructed using the Nextera XT DNA library preparation kit (Illumina, San Diego, CA, USA), followed by dual-end sequencing using Illumina NovaSeq 6000. Low-quality data were filtered using the fastp (https://github.com/OpenGene/fastp) software (version 1.0.1), and the criteria were designed to remove reads with an N base content exceeding 5%, reads with a low-quality (quality value ≤ 5) base number reaching 50%, and reads with adapter contamination, resulting in effective data (clean reads). After filtering the raw data, 7.46 G clean data was obtained (with a GC content of 41.84%, a Q20 value of 94.72% and a Q30 value of 89.03%). Subsequently, the clean data were de novo assembled using SPAdes v.3.14.1, and k-mer 21, 45, 65, 85 and 105 were set to obtain the graph file, and the graph file was visualized with the Bandage software to complete further analysis and assembly of the complete mitochondrial genome. The assembled complete mitochondrial genome was annotated using the MITOS (http://mitos.bioinf.uni-leipzig.de/index.py) software version 3.0, and the annotation results were manually corrected. Finally, we used Organellar Genome DRAW v1.2 to draw the mitochondrial genome circle diagram online. We performed next-generation sequencing to characterize the mitochondrial genome of *Cambaroides dauricus* (Supplementary Figure S1). The average sequencing depth of its mitochondrial genome was 100×. In this study, we extracted muscle tissue from *Cambaroides dauricus* and sequenced its mitochondrial genome. The genetic composition and mitochondrial genome structure arrangement of this species exhibit high similarity to those found in other *Chelidae* species.

Molecular evolution analysis was based on IQTREE and MEGA11 [24]. We first aligned the well—matched sequence files using the online MAFFT tool. Subsequently, we submitted the obtained alignment file to the IQTREE online software, with the bootstrap value set to 1000. After that, we opened the acquired file with the MEGA 11 software and perform aesthetic adjustments within MEGA. And *Procambarus clarkii* was chosen as the outgroup for phylogenetic tree construction. Phylogenetic analysis was performed with ML methods and the best-fitting nucleotide substation model with the lowest BIC score determined using the gBlocks program [25]. ML analysis were performed using the GTR + G, and the robustness of the tree topology was assessed with 1000 bootstrap replicates [26]. Additionally, there are 2 previously reported whole mitogenome sequences of *Cambaroides dauricus* at the NCBI (nucleic accession nos.: OL542521, NC_033505). We used BioEdit software (version: 11.0) to perform a comparative analysis of these sequences.

### 2.2. Amplification, Cloning and Sequencing of 5S rDNA Sequences

PCR amplification [27] and agarose gel electrophoresis detection: the study of Zhang lin and others [28] was referred to for the overall reaction system and the PCR amplification procedure and specific process, during which the PCR amplification products were subjected to agarose gel electrophoresis for detection. Rubber recycling: for the concrete steps, refer to the rubber recycling kit (Shanghai Sangong Biotech, Shanghai, China) for guidance. Connection transformation: add 2 μL of solution buffer, 2.5 μL of gel recovery product and 0.5 μL of pMD18-T vector into a 1.5 mL EP tube. Mix well and incubate at 4 °C in the refrigerator overnight for connection. Place the DH5-αEscherichia coli competent cells on ice. When they become semi-melted, take 25 μL and add it to the connected EP tube. Incubate on ice for 30 min, then activate at 42 °C for 90 s and incubate on ice for 5 min. Add 400 μL of LB liquid medium (without AMP^+^), place it in a 37 °C shaking incubator for about 1 h. Take it out and centrifuge at 6000 rpm for 2 min. On an ultra-clean workbench, use a pipette to remove 400 μL, then gently stir and disperse the precipitate with a pipette, add 100 μL of bacterial solution to LB solid medium (containing AMP^+^), use a sterilized spreader to spread it evenly and place it in the 37 °C incubator for 1 h in a forward orientation, then invert and culture for 12–18 h. Isolation: on a laminar flow bench, pick a single colony from the culture medium and transfer it to an EP tube containing 300 µL liquid culture medium (containing AMP^+^). Place the EP tube on a 37 °C shaker for 3 h of cultivation. Detection and sequencing: perform PCR on the bacterial culture, detect the products through agarose gel electrophoresis and send the results to the sequencing company for sequencing. A molecular phylogenetic tree was constructed using the neighbor-joining method (NJ), and the branch confidence was evaluated using 1000 bootstrap replications [29].

### 2.3. Determination Method of Amino Acid Content Composition

Take 2–5 g of 30 fresh *Cambaroides dauricus*’ muscles and place it at the bottom of a specially designed hydrolysis tube. Add 6 mol/L hydrochloric acid and hydrolyze it at 110 °C for 24 h. Then, determine the amino acid content using an Agilent liquid chromatograph. Detection chromatographic conditions: the separation column is C18 (4.0 mm × 125 mm) and the column temperature is 40 °C. The mobile phase A is 20 mmol/L sodium acetate, and the mobile phase B is V (20 mmol/L sodium acetate): V (methanol): V (ethylene glycol) = 1:2:2. The flow rate is 1.0 mL/min. The detection wave length of ultraviolet light is 338 nm. We used analysis of variance (ANOVA) [30] and multiple comparison tests (LSD method) [31] to test for differences in amino acid content composition using SPSS Statistics 21.0. The values of the independent variables were expressed as the mean ± SD.

## 3. Results

### 3.1. Mitochondrial Analysis

**Figure 2:**
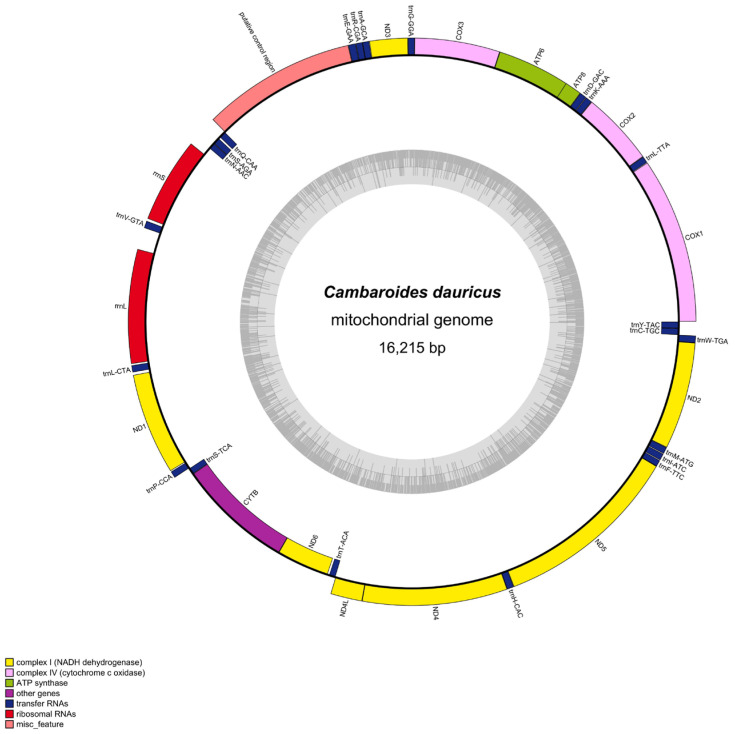
Mitochondrial genome map of *Cambaroides dauricus*.

The total mitochondrial genome of *Cambaroides dauricus* was 16,215 bp in length, which contained 2 rRNA genes (*rrnS* and *rrnL*), 13 protein-coding genes (PCGs), 22 tRNA genes and 1 putative control region (https://mitofish.aori.u-tokyo.ac.jp/annotation/input/) (8 April 2024). The complete mitochondrial genome sequence was submitted to the NCBI with GenBank number PP461738. Most genes were on the heavy strand while a few, such as the *ND6* and *CYTB* genes and 7 tRNA genes (*trnQ-CAA*, *trnS-AGA*, *trnN-AAC*, *trnS-TCA*, *trnT-ACA*, *trnC-TGC*, *trnY-TAC*) were on the light strand (Figure 2 and Table 1). The total base composition was 33.4% A, 11.0% C, 16.5% G and 39.1% T.

In the invertebrates, the start codes of protein-coding genes usually contain the codons TTG, ATT, ATC, ATA, ATG and GTG. For *Cambaroides dauricus*, most of the protein-coding genes started with the standard ATG codon. For the others, *ND1* started with the ATA codon, *ND3* and *ND6* with the ATT codon, and *ND5* with the GTG codon, while the start codon of *COX1* was not determined. The termination codons of protein-coding genes were diverse, including TAG, TAA and T. Additionally, the *ND1* was terminated in TAG. The *COX2* and *CYTB* were terminated in T. The 12S rRNA (*rrnS*, 792 bp) and 16S rRNA (*rrnL*, 1054 bp) were separated by the *trnV-GTA* gene. Between the *trnE-GAA* and the *trnQ-CAA* genes was the putative control region, which was 1424 bp in length.

**Figure 3:**
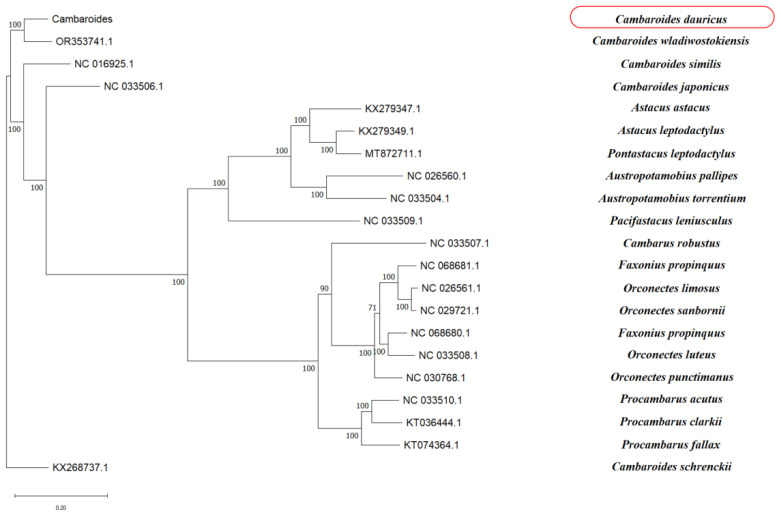
Phylogenetic tree was constructed using the Maximum-likelihood method based on whole mitogenomes of *Cambaroides dauricus* and other closely related organisms. The following sequences of the phylogenetic analysis were used: *Cambaroides dauricus* (Pallas,1772) (PP461738.1), *Cambaroides wladiwostokiensis* (OR353741.1), *Cambaroides similis* (NC_016925.1), *Cambaroides japonicus* (NC_033506.1), *Astacus astacus* isolate *Aast1* mitochondrion (KX279347.1), *Astacus leptodactylus* (KX279349.1), *Pontastacus leptodactylus* (MT872711.1), *Austropotamobius pallipes* (NC_026560.1), *Austropotamobius torrentium* (NC_033504.1), *Pacifastacus leniusculus* (NC_033509.1), *Cambarus robustus* (NC_033507.1), *Faxonius propinquus* isolate *WGL_33* mitochondrion (NC_068681.1), *Orconectes limosus* (NC_026561.1), *Orconectes sanbornii* (NC_029721.1), *Faxonius virilis* (NC_068680.1), *Orconectes luteus* (NC_033508.1), *Orconectes punctimanus* (NC_030768.1), *Procambarus acutus* (NC_033510.1), *Procambarus clarkii* (KT036444.1), *Procambarus fallax* (KT074364.1), *Cambaroides schrenckii* (KX268737.1).

The phylogenetic analysis showed that the genetic relationship between *Cambaroides dauricus* and *Cambaroides wladiwostokiensis* was the closest (Figure 3).

*Cambaroides*, *Astacus* and *Orconectes* all belong to the order *Decapoda*. *Cambaroides* is a genus of crayfish predominantly distributed in freshwater environments across North America, particularly in certain regions of the United States and Canada. *Astacus* represents a genus of crayfish in Europe, which includes several well-known species of freshwater crayfish, such as the European crayfish (*Astacus astacus*). The phylogenetic relationships between *Cambaroides* and *Astacus* are relatively close, while *Orconectes* shows a more distant relationship to both *Cambaroides* and *Astacus*, with a closer affinity to the genus *Procambarus*. *Orconectes* is another genus of crayfish native to North America, commonly referred to as North American crayfish, and encompasses several species, including *Orconectes* virilis.

Apart from the *Cambaroides* species, the rest could also form a clade, which showed the genetic biodiversity and connectivity between different species.

### 3.2. 5S rDNA Analysis

**Figure 4:**
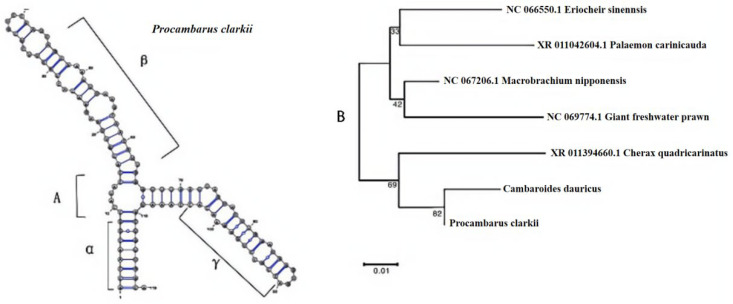
The 5S rRNA secondary structure and construction of a neighbor-joining (NJ) tree based on the 5S sequences: (**A**) *Cambaroides dauricus* 5S rRNA secondary structure; (**B**) construction of a neighbor-joining (NJ) tree based on the 5S sequence. Scale bar was 0.01.

The variations of the 5S rDNA gene mainly occur in the NTS region, while the coding region of the 5S rDNA gene shows relatively high conservation. Using the specific primers of 5S rDNA and taking the DNA of the northeastern amphipod as the template, PCR amplification was carried out, and the lengths of the obtained bands were all around 119 bp. The GC content of *Cambaroides dauricus* was 53.78%, resulting from the standard secondary structure of 5S rRNA [22]. The whole secondary structure of *Cambaroides dauricus*’ 5S rRNA gene consists of three regions (named α, β and γ), the connected hinge structure is named as Ring A. There is only one difference in a pair of base arrangements in the B ring of the β region between the two species, which indicates that the coding region of 5S rRNA has high stability. After searching for the 5S rDNA sequences of closely related species on the NCBI website, we constructed a phylogenetic tree using the MAGE software. The results are shown in Figure 4. Through the phylogenetic tree, the species can be divided into two branches. One of the branches consists of Eriocheir sinensis, Exopalaemon carinicauda, Macrobrachium nipponense, Macrobrachium rosenbergii; the other consists of *Procambarus clarkii*, *Cambaroides dauricus* and *Cherax quadricarinatus*. According to the phylogenetic tree, we can establish that the genetic relationship between *Procambarus clarkii* and *Cambaroides dauricus* is quite close.

### 3.3. Nutrition Ingredient Analysis

The determination results of *Cambaroides dauricus*’ muscles’ general nutrient components were as follows. The total amino acids measured in *Cambaroides dauricus*’ muscles were 152,144.28 mg/kg. There are 15 amino acids in its muscles’ protein, comprising 8 essential amino acids (EAA), 7 non-essential amino acids (NEAA) and proline (Pro). Among the 15 amino acids, glutamic acid, aspartic acid, alanine and glycine are umami amino acids [32].

Among the 15 kinds of amino acids measured in *Cambaroides dauricus*’ muscles, glutamic acid accounted for the highest total amount of creatine (15.6%), while histidine accounted for the lowest, at 2.05%. Glutamic acid is involved in the synthesis of various physiologically active substances and plays a detoxification role in tissues such as the brain, muscles and liver [33]. Among essential amino acids, the one with the highest content is lysine, accounting for 7.40%, and the one with the lowest content is histidine, accounting for 2.05%. Lysine is the first restricted creatine of cereal proteins and the first restricted creatine of human milk. Therefore, *Cambaroides dauricus* can precisely make up for the deficiency of lysine in cereal protein, thereby improving the utilization rate of protein. In addition, as shown in Table 2, the proportion of umami amino acids in *Cambaroides dauricus* is 36.27%. Among them, glutamic acid and aspartic acid are characteristic amino acids that present an umami taste; glycine and alanine are characteristic amino acids with a sweet taste. The presence of these umami amino acids endows *Cambaroides dauricus* with a pleasant flavor. Table 2 and Table 3 show that the ratio of essential amino acids to total creatine in the muscle of *Cambaroides dauricus* is 41.59%, and the ratio of essential amino acids to non-essential amino acids is 60.77%. According to the ideal model of FAO/WHO, for high-quality proteins, the EAA/TAA ratio of the constituent amino acids is about 40%, and the EAA/NEAA ratio is above 60%. The amino acid composition of the muscle of *Cambaroides dauricus* is superior to the requirements of the above indicators. In conclusion, *Cambaroides dauricus* is an important food source for humans to obtain amino acids and essential amino acids.

## 4. Discussion

This research described the first complete mitogenome of *Cambaroides dauricus*, providing fundamental data for relevant genetic and evolutionary studies [11]. The results showed that the complete mitogenome of *Cambaroides dauricus* was 16,215 bp in length. We chose the homologous species given by the BLAST (Version + 2.17.0) analysis of the *Cambaroides dauricus*, and concluded that *Cambaroides wladiwostokiensis* had the highest similarity with *Cambaroides dauricus*.

The experimental results of Luo et al. indicated that the mitochondrial genome sequence length of *Cambaroides dauricus* was 15,580 bp, containing 13 protein-coding genes, 22 transcriptional RNAs, and 2 ribosome genes [1]. These results are like ours, which showed that 13 proteins were similar in their mitochondrial genomes, showing significant anti-cytosine phenomena in *P. clarkii* and *C. dauricus*.

Additionally, we used BioEdit to determine that the *Cambaroides* species had higher similarity (over 85%) than other species [34]. According to previous studies, the reason that *Cambaroides japonicus*, *Cambaroides schrenckii* and *Cambaroides similis* show less similarity in the sequence alignment might be the difference in *ND5*, *ND6* and *CYTB* [6]. We think these data will contribute to the genetic conservation of *Cambaroides dauricus* and phylogenetic relationships among *Cambaroides*. This is consistent with the classification results obtained by Luo et al. [1] and Zheng et al. using a single gene, 16S rRNA [35].

The 5S rDNA is composed of the non-transcribed spacer region (NTS) and the transcribed coding sequence (CDS) [36,37]. Among them, the CDS is a highly conserved sequence. The structure of *Cambaroides dauricus*’ 5S rRNA was relatively stable. According to the phylogenetic tree constructed using 5S rDNA, we can establish that the genetic relationship between *Procambarus clarkii* and *Cambaroides dauricus* is quite close.

## 5. Conclusions

The above analysis indicated that *Cambaroides dauricus* is a kind of aquatic product with a relatively high nutritional value and a high content of protein and amino acids. Moreover, the proportion of essential amino acids in the total amino acids is relatively high, the amino acid composition is reasonable, the fat content is low and it is rich in essential fatty acids for the human body such as linoleic acid. Furthermore, due to the presence of a relatively high amount of umami amino acids and unsaturated fatty acids, *Cambaroides dauricus* has high nutrition and a pleasant flavor, and has certain beneficial health effects. Therefore, *Cambaroides dauricus*, as a freshwater aquatic product, has promising development prospects. Our research also provided essential data for relevant artificial breeding and food processing.

## Figures and Tables

**Table 1:** Characteristics of the mitochondrial genome of *Cambaroides dauricus* (“+” stands for heavy strand, and “−” stands for light strand).

Gene	Start	End	Start Code	Stop Code	Length	Strand
*COX1*	<1	1536	*/*	*TAA*	1536	+
*trnl-TTA*	1539	1602			64	+
*COX2*	1603	2287	*ATG*	*T-*	685	+
*trnk-AAA*	2288	2351			64	+
*trnd-GAC*	2353	2416			64	+
*ATP8*	2417	2575	*ATG*	*TAA*	159	+
*ATP6*	2569	3243	*ATG*	*TAA*	675	+
*COX3*	3243	4031	*ATG*	*TAA*	789	+
*trnG-GGA*	4030	4091			62	+
*ND3*	4092	4445	*ATT*	*TAA*	354	+
*trnA-GCA*	4447	4508			62	+
*trnR-CGA*	4508	4570			63	+
*trnE-GAA*	4571	4639			69	+
*putative control region*	4640	6063			1424	+
*trnQ-CAA*	6064	6132			69	−
*trnS-AGA*	6149	6215			67	−
*trnN-AAC*	6216	6279			64	−
*rrnS*	6356	7147			792	+
*trnV-GTA*	7170	7237			68	+
*rrnL*	7439	8492			1054	+
*trnL-CTA*	8506	8570			65	+
*ND1*	8595	9536	*ATA*	*TAG*	942	+
*trnP-CCA*	9544	9607			64	+
*trnS-TCA*	9611	9674			64	−
*CYTB*	9675	10,809	*ATG*	*T-*	1135	−
*ND6*	10,809	11,327	*ATT*	*TAA*	519	−
*trnT-ACA*	11,345	11,407			63	−
*ND4L*	11,410	11,703	*ATG*	*TAA*	294	+
*ND4*	11,703	13,403	*ATG*	*TAA*	1341	+
*trnH-CAC*	13,043	13,106			64	+
*ND5*	13,107	14,837	*GTG*	*TAA*	1731	+
*trnF-TTC*	14,837	14,897			61	+
*trnI-ATC*	14,901	14,964			64	+
*trnM-ATG*	14,968	15,030			63	+
*ND2*	15,031	16,023	*ATG*	*TAA*	993	+
*trnW-TGA*	16,023	16,088			66	+
*trnC-TGC*	16,088	16,152			65	−
*trnY-TAC*	16,152	16,215			64	−

**Table 2:** Content distribution of amino acids.

Amino Acids	Content	Amino Acids	Content
aspartic acid	10.31	lysine	7.40%
glutamic acid	15.61	isoleucine	2.88%
glycine	4.44	leucine	6.94%
alanine	5.91	threonine	3.34%
serine	3.96	phenylalanine	3.63%
proline	3.94	total amino acids (TAA)	152,144.28 mg/kg
tyrosine	3.29	total essential amino acids (EAA)	41,881.16 mg/kg
arginine	10.18	total umami amino acids	55,178.64 mg/kg
histidine	2.05	EAA/TAA × 100	41.59%
valine	3.33	EAA/NEAA × 100	60.77%

**Table 3:** Distribution of moisture, crude fat, crude protein and crude ash content.

Species Name	Moisture Content (mg/100 mg)	Crude Fat Content (mg/100 mg)	Crude Protein Content (mg/100 mg)	Crude Ash Content (mg/100 mg)
*Cambaroides dauricus*	79.8 ± 0.72	0.83 ± 0.12	18.47 ± 0.87	0.63 ± 0.06

## Data Availability

The genome sequence data that support the findings of this study are openly available in GenBank of NCBI at (https://www.ncbi.nlm.nih.gov/) under the accession no. PP461738. The associated BioProject, SRA, and BioSample numbers are PRJNA918546, SRS16363390, and SAMN32595059, respectively.

## Supplementary Materials

The following supporting information can be downloaded at: https://www.mdpi.com/article/10.3390/biology14091215/s1, Figure S1: The coverage depth map of *Cambaroides dauricus* high-throughput DNA sequencing.
