# Supplementary figures and images for: Research on 5S rDNA, Mitochondria and Nutritional Components of Cambaroides dauricus
Source: Biology (Basel). 2025 Sep 8;14(9):1215. doi: 10.3390/biology14091215 (PMC12467288; doi:10.3390/biology14091215)

**Figure S1.** The coverage depth map of *Cambaroides dauricus* high-throughput DNA sequencing.

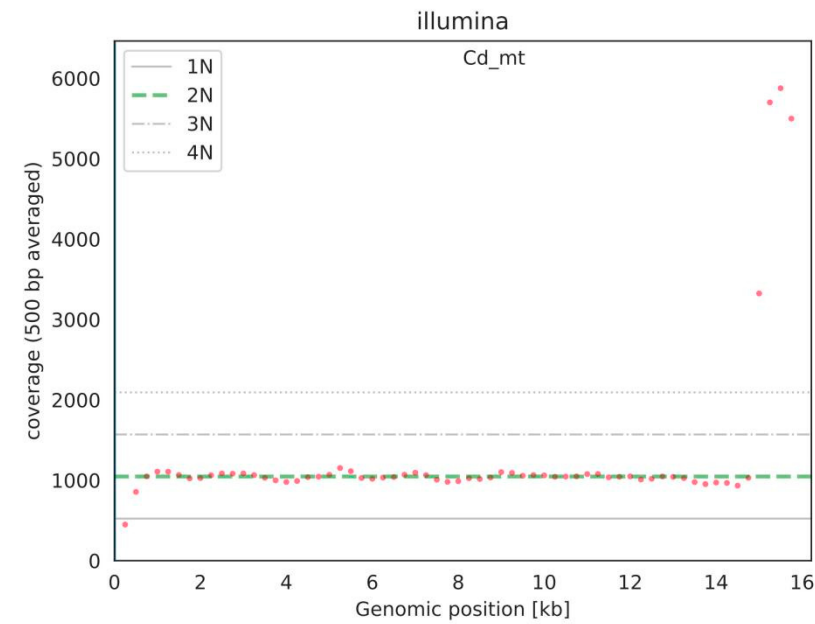

Supplement: Supplementary file 1 [file biology-14-01215-s001.zip › biology-3735805-supplementary.pdf]
